# On Frame’s inequalities

**DOI:** 10.1186/s13660-018-1687-x

**Published:** 2018-04-20

**Authors:** Ling Zhu

**Affiliations:** Department of Mathematics, Zhejiang Gongshang University, Hangzhou, China

**Keywords:** 26D05, 26D15, 33B10, Circular functions, Hyperbolic functions, Frame’s inequalities, Cusa–Huygens inequalities

## Abstract

In this paper, the errors of the two inequalities in Theorem 3.4.20 in the classic “Analytic Inequalities” by Mitrinovic are corrected, and the corresponding inequalities for circular functions and hyperbolic functions are rebuilt.

## Introduction

The classic “Analytic Inequalities” by Mitrinovic [1] has been hailed all over the world since it was published in 1970. The influence of this book on the various branches of mathematics cannot be overestimated and will last forever. As the author says in the introduction of his book, “The greater part of the results included have been checked, although this could not, of course, be done for all the results which appear in the book. We hope, however, that there are not many errors, but the very nature of this book is such that it seems impossible to expect it to be entirely free of them.” I find some errors in “Analytic Inequalities” and announce the specific contents.

The following conclusions are on p. 241 in [1].

### Proposition 1

([1, Theorem 3.4.20])

*For every* $t>0$,

1.1 $$\begin{aligned}& t-\frac{3\sin t}{2+\cos t}< \frac{1}{180}t^{5}, \end{aligned}$$

1.2 $$\begin{aligned}& t-\frac{3\sin t}{2+\cos t} \biggl( 1+ \frac{(1-\cos t)^{2}}{9(3+2\cos t)} \biggr) < \frac{t^{7}}{2100}. \end{aligned}$$

It is not difficult to find that these two inequalities above on the common interval $(0,\pi /2)$ are wrong. I read carefully the only one citation [2] by Frame in [1] for Theorem 3.4.20, which was published in the 1944 issue of “The American Mathematical Monthly” in the form of the report of the Mathematical Seminar. We judge that the object of Frame [2] is a right triangle, so the *t* must be the other two acute angles of a right triangle, that is, $t\in (0,\pi /2)$. We can only find the related contents of (1.1) in [2] is the item “(7)”, but the one (1.2) at least did not appear in [2].

By using the analytic method, this paper has come to the corresponding conclusions of (1.1) and (1.2); specifically, these are, in the form of (1.1) and (1.2), the first of two inequalities holds for hyperbolic functions, while the second one must be reconstructed, and reversely for circular functions on the interval $(0,\pi )$.

### Theorem 1

*Let* $x\in (0,\pi )$. *Then*

1.3 $$ x-\frac{3\sin x}{2+\cos x}> \frac{1}{180}x^{5} $$

*and*

1.4 $$ x-\frac{3\sin x}{2+\cos x} \biggl( 1+ \frac{(1-\cos x)^{2}}{9(3+2\cos x)} \biggr) >\frac{x^{7}}{2100} $$

*hold*, $1/180$ *and* $1/2100$ *are the best constants in* (1.3) *and* (1.4), *respectively*.

### Theorem 2

*Let* $x>0$. *Then*

1.5 $$ x-\frac{3\sinh x}{2+\cosh x}< \frac{1}{180}x^{5} $$

*and*

1.6 $$ x-\frac{3\sinh x}{2+\cosh x} \biggl( 1+\frac{(1-\cosh x)^{2}}{9(3+2 \cosh x)} \biggr) < - \bigl( 9.537610179\cdot 10^{-5} \bigr) x^{7} $$

*hold*, *where* $1/180$ *is the best constant in* (1.5).

## Lemmas

### Lemma 1

(Mitrinovic–Adamovic inequality [3])

*The inequality*

2.1 $$ \biggl( \frac{\sin t}{t} \biggr) ^{3}>\cos t $$

*holds for all* $t\in (0,\pi /2)$, *and the exponent* 3 *is best possible*.

### Lemma 2

(Lazarevic’s inequality [4])

*Let* $t\neq 0$. *Then*

2.2 $$ \biggl( \frac{\sinh t}{t} \biggr) ^{3}>\cosh t $$

*holds*, *and the exponent* 3 *is best possible*.

### Lemma 3

([5])

*Let* $\vert x\vert <\pi $, *and* $B_{2n}$ *be the even*-*indexed Bernoulli numbers* (*see* [6]), *we have the following power series expansion*:

2.3 $$ \frac{1}{\sin x}=\frac{1}{x}+\sum_{n=1}^{\infty } \frac{2^{2n}-2}{ ( 2n ) !}\vert B_{2n} \vert x^{2n-1}.$$

### Lemma 4

([7])

*Let* $a_{n}$ *and* $b_{n}$ ($n=0,1,2,\ldots$) *be real numbers*, *and let the power series* $A(t)=\sum_{n=0}^{\infty }a_{n}t^{n}$ *and* $B(t)=\sum_{n=0}^{\infty }b_{n}t^{n}$ *be convergent for* $\vert t\vert < R$ ($R\leq +\infty $). *If* $b_{n}>0$ *for* $n=0,1,2,\ldots$ , *and if* $\varepsilon_{n}=a_{n}/b_{n}$ *is strictly increasing* (*or decreasing*) *for* $n=0,1,2,\ldots$ , *then the function* $A(t)/B(t)$ *is strictly increasing* (*or decreasing*) *on* $(0,R)$ ($R\leq +\infty $).

### Lemma 5

([8])

*Let* $B_{2n}$ *be the even*-*indexed Bernoulli numbers*. *Then the double inequality*

2.4 $$ \frac{\pi^{2}(2^{2n+2}-1)}{(2n+2)(2n+1)(2^{2n}-1)}< \frac{\vert B _{2n}\vert }{\vert B_{2n+2}\vert }< \frac{\pi^{2}(2^{2n+1}-1)}{(2n+2)(2n+1)(2^{2n-1}-1)} $$

*holds for* $n=1,2,\ldots $ .

### Lemma 6

*Let* $B_{2n}$ *be the even*-*indexed Bernoulli numbers*. *Then the series*

2.5 $$ \{ \delta_{n} \} = \biggl\{ \frac{n(2^{2n+2}-2)\vert B_{2n+2} \vert }{(2n+2)(2^{2n}-2)\vert B_{2n} \vert } \biggr\} $$

*is increasing for* $n\geq 1$.

### Proof

By Lemma 5 we have

$$\begin{aligned}& \begin{aligned} \delta_{n-1} &= \frac{(n-1)(2^{2n}-2)\vert B_{2n} \vert }{(2n)(2^{2n-2}-2)\vert B_{2n-2} \vert } \\ &< \frac{(n-1)(2^{2n}-2)}{(2n)(2^{2n-2}-2)}\frac{(2n)(2n-1)(2^{2n-2}-1)}{\pi^{2}(2^{2n}-1)}, \end{aligned} \\& \begin{aligned} \delta_{n} &= \frac{n(2^{2n+2}-2)\vert B_{2n+2} \vert }{(2n+2)(2^{2n}-2)\vert B_{2n} \vert } \\ &>\frac{n(2^{2n+2}-2)}{(2n+2)(2^{2n}-2)}\frac{(2n+2)(2n+1)(2^{2n-1}-1)}{ \pi^{2}(2^{2n+1}-1)}. \end{aligned} \end{aligned}$$

In order to prove $c_{n-1}< c_{n}$ for $n\geq 2$ it suffices to show

$$\begin{aligned} &\frac{n(2^{2n+2}-2)}{(2n+2)(2^{2n}-2)}\frac{(2n+2)(2n+1)(2^{2n-1}-1)}{ \pi^{2}(2^{2n+1}-1)} \\ &\quad >\frac{(n-1)(2^{2n}-2)}{(2n)(2^{2n-2}-2)}\frac{(2n)(2n-1)(2^{2n-2}-1)}{ \pi^{2}(2^{2n}-1)}, \end{aligned}$$

that is,

2.6 $$ 2n(2n+1) \bigl(2^{2n}-8\bigr) \bigl(2^{2n}-1\bigr)>(2n-2) (2n-1) \bigl(2^{2n}-4\bigr) \bigl(2^{2n}-2\bigr). $$

Let $2n=m$. Then, for $m\geq 4$, we show

2.7 $$ m(m+1) \bigl(2^{m}-8\bigr) \bigl(2^{m}-1\bigr)>(m-2) (m-1) \bigl(2^{m}-4\bigr) \bigl(2^{m}-2\bigr). $$

Let

$$\begin{aligned}& u_{m} =m(m+1) \bigl(2^{m}-8\bigr) \bigl(2^{m}-1 \bigr)-(m-2) (m-1) \bigl(2^{m}-4\bigr) \bigl(2^{m}-2\bigr) \\& \hphantom{u_{m}}=2 (2m-1)2^{2m}-\bigl(3m^{2}+27m-12 \bigr)2^{m}+32m- 16, \\& v_{m} =2\cdot 2^{m}-\frac{3m^{2}+27m-12}{2m-1}. \end{aligned}$$

Then

2.8 $$\begin{aligned} u_{m} &>2 (2m-1)2^{2m}-\bigl(3m^{2}+27m-12 \bigr)2^{m} \\ &= (2m-1)2^{m}v_{m} \end{aligned}$$

and

2.9 $$ v_{m+1}-2v_{m}= 3\cdot \frac{17m^{2}+2m^{3}+m-2}{ ( 2m-1 ) ( 2m+1 ) }>0 $$

for $m\geq 4$.

It follows from (2.9) and $v_{4}=80/7>0$ that $v_{m}>0$ for $m\geq 4$. Therefore, Lemma 6 follows from (2.6), (2.7) and (2.8). □

### Lemma 7

*The function*

$$ K(t)=\frac{t^{3}-\sin^{3}t}{t^{3}\sin^{2}t} $$

*is increasing on* $(0,\pi )$. *In particular*, *we have*

(i) *The double inequality*
  2.10 $$ 1-\frac{\pi^{3}-8}{\pi^{3}}\sin^{2}t< \biggl( \frac{\sin t}{t} \biggr) ^{3}< 1-\frac{1}{2}\sin^{2}t $$
  *holds for all* $t\in (0,\pi /2)$, *the constants* $(\pi^{3}-8)/\pi^{3}$ *and* $1/2$ *are best possible*.
(ii) *The inequality*
  2.11 $$ \biggl( \frac{\sin t}{t} \biggr) ^{3}< 1-\frac{\pi^{3}-8}{\pi^{3}}\sin ^{2}t $$
  *holds for all* $t\in (\pi /2,\pi )$, *and the constant* $(\pi^{3}-8)/\pi ^{3}$ *is best possible*.

### Proof

Let

$$ K(t)=\frac{t^{3}-\sin^{3}t}{t^{3}\sin^{2}t}=\frac{ ( \frac{t}{ \sin t} ) ^{3}-1}{\frac{t^{3}}{\sin t}}:=\frac{A(t)}{B(t)},\quad 0< t< \pi . $$

Then by (2.3) we obtain

$$\begin{aligned} \frac{1}{\sin^{3}t} &=\frac{1}{\sin t}+\frac{1}{2} \biggl( \frac{1}{ \sin t} \biggr) ^{\prime \prime } \\ &=\frac{1}{t}+\sum_{n=1}^{\infty } \frac{2^{2n}-2}{ ( 2n ) !}\vert B_{2n} \vert t^{2n-1} \\ &\quad {}+\frac{1}{t^{3}}+\sum_{n=2}^{\infty } \frac{(2^{2n}-2)(2n-1)(n-1)}{ ( 2n ) !}\vert B_{2n} \vert t^{2n-3} \end{aligned}$$

and

$$\begin{aligned}& \begin{aligned} A(t) &=t^{3} \Biggl( \frac{1}{t}+\sum _{n=1}^{\infty }\frac{2^{2n}-2}{ ( 2n ) !}\vert B_{2n} \vert t^{2n-1} \Biggr) -1 \\ &\quad {}+t^{3} \Biggl( \frac{1}{t^{3}}+\sum_{n=2}^{\infty } \frac{(2^{2n}-2)(2n-1)(n-1)}{ ( 2n ) !}\vert B_{2n} \vert t^{2n-3} \Biggr) \\ &=t^{2}+\sum_{n=1}^{\infty } \biggl[ \frac{(2^{2n}-2)\vert B_{2n} \vert }{ ( 2n ) !}+ \frac{(2^{2n+2}-2)(2n+1)n\vert B_{2n+2} \vert }{ ( 2n+2 ) !} \biggr] t ^{2n+2} \\ &:=\sum_{n=1}^{\infty }a_{n}t^{2n+2}, \end{aligned} \\& \begin{aligned} B(t) &=t^{3} \Biggl( \frac{1}{t}+\sum _{n=1}^{\infty }\frac{2^{2n}-2}{ ( 2n ) !}\vert B_{2n} \vert t^{2n-1} \Biggr) =t^{2}+\sum _{n=1}^{\infty }\frac{(2^{2n}-2)\vert B_{2n} \vert }{ ( 2n ) !}t^{2n+2} \\ &:=\sum_{n=0}^{\infty }b_{n}t^{2n+2}, \end{aligned} \end{aligned}$$

where

$$\begin{aligned}& a_{0} = 1,\qquad a_{n}=\frac{(2^{2n}-2)\vert B_{2n} \vert }{ ( 2n ) !}+\frac{(2^{2n+2}-2)(2n+1)n\vert B_{2n+2} \vert }{ ( 2n+2 ) !}, \\& b_{0} = 1,\qquad b_{n}=\frac{(2^{2n}-2)\vert B_{2n} \vert }{ ( 2n ) !}>0,\quad n \geq 1. \end{aligned}$$

Since

$$ \frac{a_{0}}{b_{0}}=1,\qquad \frac{a_{n}}{b_{n}}=1+\frac{n(2^{2n+2}-2)\vert B_{2n+2} \vert }{(2n+2)(2^{2n}-2)\vert B_{2n} \vert }=1+ \delta_{n},\quad n\geq 1, $$

we know $a_{0}/b_{0}< a_{1}/b_{1}$, and $\{ a_{n}/b_{n} \} _{n\geq 1}$ is increasing by Lemma 6. So $\{ a_{n}/b_{n} \} _{n\geq 0}$ is increasing, and $K(t)=A(t)/B(t)$ is increasing on $(0,\pi )$ by Lemma 4. In view of

$$ K\bigl(0^{+}\bigr)=\frac{1}{2},\qquad K \biggl( \frac{\pi }{2} \biggr) =\frac{\pi^{3}-8}{ \pi^{3}},\qquad K\bigl(\pi^{-}\bigr)=+\infty, $$

this completes the proof of Lemma 6. □

In order to prove (1.6), we need the following lemmas. We introduce a useful auxiliary function $H_{f,g}$. For $-\infty \leq a< b\leq \infty $, let *f* and *g* be differentiable on $(a,b)$ and $g^{\prime }\neq 0$ on $(a,b)$. Then the function $H_{f,g}$ is defined by

$$ H_{f,g}:=\frac{f^{\prime }}{g^{\prime }}g-f. $$

The function $H_{f,g}$ has some good properties and plays an important role in the proof of a monotonicity criterion for the quotient of power series.

### Lemma 8

([9])

*Let* $C ( t ) =\sum_{k=0}^{\infty }c_{k}t^{k}$ *and* $D ( t ) =\sum_{k=0}^{\infty }d_{k}t^{k}$ *be two real power series converging on* $( -r,r ) $ ($r\leq +\infty $) *and* $d_{k}>0$ *for all k*. *Suppose that*, *for certain* $m\in \mathbb{N} $, *the non*-*constant sequence* $\{ a_{k}/b_{k} \} $ *is increasing* (*resp*. *decreasing*) *for* $0\leq k\leq m$ *and decreasing* (*resp*. *increasing*) *for* $k\geq m$. *Then the function* $C/D$ *is strictly increasing* (*resp*. *decreasing*) *on* $( 0,r ) $ *if and only if* $H_{C,D} ( r^{-} ) \geq $ (*resp*. ≤) 0. *Moreover*, *if* $H_{C,D} ( r^{-} ) <$ (*resp*. >) 0, *then there exists* $t_{0}\in ( 0,r ) $ *such that the function* $C/D$ *is strictly increasing* (*resp*. *decreasing*) *on* $( 0,t_{0} ) $ *and strictly decreasing* (*resp*. *increasing*) *on* $( t_{0},r ) $.

### Lemma 9

*Let*

$$ L(t)=\frac{\sinh^{3}t-t^{3}}{t^{3}\sinh^{2}t},\quad t>0, $$

*Then the function* $L(t)$ *has a minimum point* $t_{0}=2.72078\ldots $ , *and*

$$ \frac{\sinh^{3}t-t^{3}}{t^{3}\sinh^{2}t}\geq L(t_{0})=0.35803\ldots :=\theta . $$

*In particular*, *we see that the double inequality*

2.12 $$ \biggl( \frac{\sinh t}{t} \biggr) ^{3}\geq 1+\theta \sinh^{2}t $$

*holds for all* $t\in (0,+\infty )$, *the constant* *θ* *is best possible*.

### Proof

Let

$$ L(t)=\frac{ ( \frac{\sinh t}{t} ) ^{3}-1}{\sinh^{2}t}=\frac{ \sinh^{3}t-t^{3}}{t^{3}\sinh^{2}t}:=\frac{C(t)}{D(t)},\quad 0< t< +\infty . $$

Then by using the infinite series of sinh*x* and cosh*x* we obtain

$$\begin{aligned}& C(t) =\sinh^{3}t-t^{3}=\frac{1}{4} ( \sinh 3t-3 \sinh t ) -t ^{3}=\sum_{n=2}^{\infty }\frac{3^{2n+1}-3}{4 ( 2n+1 ) !}t^{2n+1}:= \sum_{n=2}^{\infty }c_{n}t^{2n+1} \end{aligned}$$

and

$$\begin{aligned} D(t) =&t^{3}\sinh^{2}t=\frac{1}{2} ( \cosh 2t-1 ) t^{3} \\ =&\sum_{n=2}^{\infty }\frac{2^{2n-3}}{ ( 2n-2 ) !}t^{2n+1}:= \sum_{n=2}^{\infty }d_{n}t^{2n+1}, \end{aligned}$$

where

$$ c_{n}=\frac{3^{2n+1}-3}{4 ( 2n+1 ) !},\qquad d_{n}=\frac{2^{2n-3}}{ ( 2n-2 ) !}>0,\quad n\geq 2. $$

Setting

$$ \zeta_{n}:=\frac{c_{n}}{d_{n}}=\frac{3^{2n+1}-3}{ ( 2n+1 ) (2n)(2n-1)2^{2n-1}},\quad n \geq 2, $$

we have

$$ \zeta_{2}=\frac{1}{2}>\zeta_{3}=\frac{13}{40}>\zeta_{4}= \frac{205}{672}, $$

and $\{ \zeta_{n} \} _{n\geq 4}$ is increasing since

$$ \zeta_{n+1}-\zeta_{n}=\frac{3}{4}\frac{ ( 10n^{2}-29n-12 ) 3^{2n}+6n ^{2}+21n+12}{2^{2n}n ( 2n+3 ) ( 2n+1 ) ( 2n-1 ) ( n+1 ) } >0 $$

for $n\geq 4$. So

$$ \zeta_{2}>\zeta_{3}>\zeta_{4}< \zeta_{5}< \zeta_{6}< \cdots . $$

We compute

$$ H_{C,D} ( +\infty ) =\lim_{x\rightarrow +\infty } \biggl( \frac{C ^{\prime }}{D^{\prime }}D-C \biggr) =+\infty, $$

and we find that there exists $t_{0}\in ( 0,+\infty ) $ such that the function $C/D$ is strictly decreasing on $( 0,t_{0} ) $ and strictly increasing on $( t_{0},+\infty ) $ by Lemma 8. Let $t_{1}=2.72078,t_{2}=2.72079$. We calculate

$$\begin{aligned}& L^{\prime }(t_{1})=-3.9522\times10^{-7} < 0, \\& L^{\prime }(t_{2})=3.7644\times 10^{-7}>0, \end{aligned}$$

and see that there exists $t_{0}\in ( t_{1},t_{2} ) = ( 2.72078,2.72079 ) \subset ( 0,+\infty ) $ such that $L^{\prime }(t_{0})=0$. So

$$ L(t_{0})=\min_{t\in (0,+\infty )}L(t)=\min_{t\in (2.72078,2.72079)}L(t), $$

and

$$ L(t_{0})\geq L(t_{2})+(t_{0}-t_{2})L^{\prime }(t_{2})= 0.35803\ldots. $$

Obviously, $L(t)\geq L(t_{0})$ implies (2.12). □

## The proof of Theorem 1

### The proof of the inequality (1.3)

Let

$$ F(x)= x-\frac{3\sin x}{2+\cos x}- \frac{1}{180}x^{5},\quad 0< x< \pi . $$

Then

$$\begin{aligned} F^{\prime }(x) &=-\frac{1}{36 ( \cos x+2 ) ^{2}} \bigl( x^{4} ( \cos x+2 ) ^{2}-36 ( 1-\cos x ) ^{2} \bigr) \\ &=-\frac{x^{2} ( \cos x+2 ) +6 ( 1-\cos x ) }{36 ( \cos x+2 ) ^{2}} \bigl[ x^{2} ( \cos x+2 ) -6 ( 1-\cos x ) \bigr] . \end{aligned}$$

In order to prove $F^{\prime }(x)>0$ holds for $x\in (0,\pi )$, it suffices to show

3.1 $$ x^{2} ( \cos x+2 ) -6 ( 1-\cos x ) < 0,\quad 0< x< \pi . $$

Since

3.2 $$ \cos x=-\frac{\tan^{2}\frac{x}{2}-1}{\tan^{2}\frac{x}{2}+1} $$

we have

3.3 $$ x^{2} ( \cos x+2 ) -6 ( 1-\cos x ) < 0\quad \Longleftrightarrow \quad 1+ \frac{3}{ ( \tan \frac{x}{2} ) ^{2}}< \frac{3}{ ( \frac{x}{2} ) ^{2}}. $$

Let $x/2=t$. Then (3.3) is equivalent to

3.4 $$ 1+\frac{3}{\tan^{2}t}< \frac{3}{t^{2}},\quad 0< t< \frac{\pi }{2}. $$

In fact, when letting

3.5 $$ f(t)=\frac{3}{t^{2}}-1-\frac{3}{\tan^{2}t},\quad 0< t< \frac{\pi }{2}, $$

then

3.6 $$ f\bigl(0^{+}\bigr)=1,f \biggl( \biggl( \frac{\pi }{2} \biggr) ^{-} \biggr) = \frac{12}{\pi^{2}}-1\thickapprox 0.21585>0. $$

By Lemma 1, we have

3.7 $$ f^{\prime }(t)=-\frac{6}{\sin^{3}t} \biggl[ \biggl( \frac{\sin t}{t} \biggr) ^{3}-\cos t \biggr] < 0,\quad 0< t< \frac{\pi }{2}, $$

which implies

3.8 $$ f(t)\geq \min_{t\in (0,\pi /2)}f(t)=f \biggl( \biggl( \frac{\pi }{2} \biggr) ^{-} \biggr) =\frac{12}{\pi^{2}}-1>0,\quad 0< t< \frac{\pi }{2}. $$

So $F^{\prime }(x)>0$ holds for $x\in (0,\pi )$, and

3.9 $$ F(x)= x-\frac{3\sin x}{2+\cos x}- \frac{1}{180}x^{5}>F(0)=0,\quad 0< x< \pi . $$

Since

$$ \lim_{x\rightarrow 0^{+}}\frac{x-\frac{3\sin x}{2+\cos x}}{x^{5}}= \frac{1}{180}, $$

this completes the proof of the inequality (1.3). □

### The proof of the inequality (1.4)

Let

$$ G(x)=x-\frac{3\sin x}{2+\cos x} \biggl( 1+\frac{(1-\cos x)^{2}}{9(3+2 \cos x)} \biggr) - \frac{x^{7}}{2100},\quad 0< x< \pi . $$

Then $G(0^{+})=0$, and

3.10 $$ G^{\prime }(x)=-\frac{1}{300 ( 2\cos x+3 ) ^{2}} \bigl[ x^{6} ( 2\cos x+3 ) ^{2}- 200 ( 1-\cos x ) ^{3} \bigr] . $$

In order to prove $G^{\prime }(x)>0$ holds for $x\in (0,\pi )$, it suffices to prove

3.11 $$ x^{6}< 200\frac{ ( 1-\cos x ) ^{3}}{ ( 2\cos x+3 ) ^{2}},\quad 0< x< \pi . $$

Via (3.2) we have

$$\begin{aligned}& 2\cos x+3=\frac{\tan^{2}\frac{x}{2}+5}{\tan^{2}\frac{x}{2}+1},\qquad 1- \cos x= 2 \frac{\tan^{2}\frac{x}{2}}{\tan^{2}\frac{x}{2}+1}, \end{aligned}$$

and (3.11) is equivalent to

3.12 $$ x^{6}< \biggl( \frac{40\tan^{3}\frac{x}{2}}{(\sec \frac{x}{2})(\sec^{2} \frac{x}{2}+4)} \biggr) ^{2}. $$

So (3.12) holds for $x\in (0,\pi )$ when proving

3.13 $$ x^{3}< \frac{40\tan^{3}\frac{x}{2}}{(\sec \frac{x}{2})(\sec^{2} \frac{x}{2}+4)},\quad 0< x< \pi, $$

or

3.14 $$ \biggl( \frac{x}{2} \biggr) ^{3}< \frac{5\tan^{3}\frac{x}{2}}{(\sec \frac{x}{2})(\sec^{2}\frac{x}{2}+4)},\quad 0< x< \pi . $$

Let $x/2=t$. Then $t\in (0,\pi /2)$, and (3.14) is equivalent to

3.15 $$ t^{3}< \frac{5\tan^{3}t}{(\sec t)(\sec^{2}t+4)}= \frac{5\sin^{3}t}{4\cos^{2}t+1}, $$

or

3.16 $$ \biggl( \frac{\sin t}{t} \biggr) ^{3}>\frac{4\cos^{2}t+1}{5}=1- \frac{4}{5}\sin^{2}t. $$

In fact, by Lemma 7 we have

3.17 $$ \biggl( \frac{\sin t}{t} \biggr) ^{3}>1-\frac{\pi^{3}-8}{\pi^{3}} \sin^{2}t>1- \frac{4}{5}\sin^{2}t $$

for all $t\in (0,\pi /2)$ due to $4/5>(\pi^{3}-8)/\pi^{3}=0.74199\ldots$ .

Since

$$ \lim_{x\rightarrow 0^{+}}\frac{x-\frac{3\sin x}{2+\cos x} ( 1+\frac{(1- \cos x)^{2}}{9(3+2\cos x)} ) }{x^{7}}= \frac{1}{2100}, $$

this completes the proof of the inequality (1.4). □

So the proof of Theorem 1 is complete.

## The proof of Theorem 2

### The proof of the inequality (1.5)

Let

$$ S(x)=x-\frac{3\sinh x}{2+\cosh x}- \frac{1}{180}x^{5},\quad 0< x< + \infty . $$

Then

$$\begin{aligned} S^{\prime }(x)&=-\frac{x^{4} ( \cosh x+2 ) ^{2}-36 ( \cosh x-1 ) ^{2}}{36 ( \cosh x+2 ) ^{2}} \\ &=-\frac{x^{2} ( \cosh x+2 ) +6 ( \cosh x-1 ) }{36 ( \cos x+2 ) ^{2}} \bigl[ x^{2} ( \cosh x+2 ) -6 ( \cosh x-1 ) \bigr]. \end{aligned}$$

In order to prove that $S^{\prime }(x)<0$ holds for $x\in (0,+\infty )$, it suffices to show

4.1 $$ x^{2} ( \cosh x+2 ) -6 ( \cosh x-1 ) >0,\quad 0< x< + \infty . $$

Since

4.2 $$ \cosh x=-\frac{\tanh^{2}\frac{x}{2}+1}{\tanh^{2}\frac{x}{2}-1} $$

we have

4.3 $$ x^{2} ( \cosh x+2 ) -6 ( \cosh x-1 ) >0\quad \Longleftrightarrow \quad 1+ \frac{3}{ ( \tanh \frac{x}{2} ) ^{2}}>\frac{3}{ ( \frac{x}{2} ) ^{2}}. $$

Let $x/2=t$. Then (4.3) is equivalent to

4.4 $$ 1+\frac{3}{\tanh^{2}t}>\frac{3}{t^{2}},\quad 0< t< +\infty . $$

In fact, when letting

4.5 $$ s(t)=\frac{3}{\tanh^{2}t}-1-\frac{3}{t^{2}},\quad 0< t< +\infty, $$

we have

4.6 $$ s\bigl(0^{+}\bigr)=1,\qquad s ( +\infty ) =2. $$

By Lemma 2 we can obtain

4.7 $$ s^{\prime }(t)=\frac{6}{\sinh^{3}t} \biggl( \biggl( \frac{\sinh t}{t} \biggr) ^{3}-\cosh t \biggr) >0,\quad 0< t< +\infty, $$

which implies

4.8 $$ s(t)\geq \min_{t\in (0,+\infty )}s(t)=s\bigl(0^{+} \bigr)=1>0,\quad 0< t< +\infty . $$

So $S^{\prime }(x)<0$ holds for $x\in (0,+\infty )$, and

4.9 $$ S(x)= x-\frac{3\sinh x}{2+\cosh x}- \frac{1}{180}x^{5}< S(0)=0,\quad 0< x< + \infty . $$

Since

$$ \lim_{x\rightarrow 0^{+}}\frac{x-\frac{3\sinh x}{2+\cosh x}}{x^{5}}= \frac{1}{180}, $$

this completes the proof of the inequality (1.5). □

### The proof of the inequality (1.6)

Let $p=9.537610179\cdot 10^{-5}$, and

$$ H(x)=x-\frac{3\sinh x}{2+\cosh x} \biggl( 1+\frac{(1-\cosh x)^{2}}{9(3+2 \cosh x)} \biggr) +px^{7},\quad 0< x< +\infty . $$

Then $H(0^{+})=0$, and

$$ H^{\prime }(x)=\frac{21px^{6} ( 2\cosh x+3 ) ^{2}-2 ( \cosh x-1 ) ^{3}}{3 ( 2\cosh x+3 ) ^{2}}. $$

We have

$$\begin{aligned}& H^{\prime }(x)>0 \quad \Longleftrightarrow \quad p< \frac{2 ( \cosh x-1 ) ^{3}}{21x^{6} ( 2\cosh x+3 ) ^{2}} \\& \hphantom{H^{\prime }(x)>0 }\quad \Longleftrightarrow \quad x^{6}< \frac{998.553028 ( \cosh x-1 ) ^{3}}{ ( 2\cosh x+3 ) ^{2}} \\& \hphantom{H^{\prime }(x)>0 \quad \Longleftrightarrow \quad x^{6}}=\frac{998.553028 ( 2\frac{\tanh^{2}\frac{x}{2}}{1-\tanh^{2}\frac{x}{2}} ) ^{3}}{ ( \frac{5-\tanh^{2}\frac{x}{2}}{1-\tanh^{2}\frac{x}{2}} ) ^{2}} =\frac{7988.424224\cosh^{2}\frac{x}{2}\tanh^{6}\frac{x}{2}}{ ( 5-\tanh^{2} \frac{x}{2} ) ^{2}} \\& \hphantom{H^{\prime }(x)>0 } \quad \Longleftrightarrow \quad x^{3}< 89.37798512 \frac{\cosh \frac{x}{2}\tanh^{3}\frac{x}{2}}{5-\tanh^{2}\frac{x}{2}} = 89.37798512\frac{\sinh^{3}\frac{x}{2}}{5\cosh^{2}x-\sinh^{2}\frac{x}{2}} \\& \hphantom{H^{\prime }(x)>0 }\quad \Longleftrightarrow \quad \biggl( \frac{\sin t}{t} \biggr) ^{3}> \frac{5+4\sinh^{2}t}{11.17224814}, \end{aligned}$$

where $t=x/2>0$. In fact, by (2.12) in Lemma 9 we have

$$ \biggl( \frac{\sinh t}{t} \biggr) ^{3}>1+0.35803\sinh^{2}t> \frac{5+4\sinh^{2}t}{11.17224814}. $$

The last inequality holds for $t\in ( 0,+\infty ) $ due to

$$\begin{aligned} & 11.17224814\bigl(1+0.35803\sinh^{2}t\bigr)-\bigl(5+4 \sinh^{2}t\bigr) \\ &\quad = 1.5642\times 10^{-9}\cosh 2t+ 6.17224814>0. \end{aligned}$$

Therefore $H^{\prime }(x)>0$, and $H(x)>H(0^{+})=0$ holds for $x\in ( 0,+\infty ) $.

So the proof of Theorem 2 is complete. □

## Remarks

### Remark 1

The inequalities (1.3) and (1.4) are obviously better than the famous Cusa–Huygens inequality (see [10–13]):

5.1 $$ \frac{\sin x}{x}< \frac{2+\cos x}{3},\quad 0< x< \frac{\pi }{2}. $$

### Remark 2

Mortici [14] strengthened (5.1) to

5.2 $$ \frac{\sin x}{x}< \frac{2+\cos x}{3}-\frac{1}{180}x^{4}+ \frac{1}{3780}x ^{6},\quad 0< x< \frac{\pi }{2}. $$

It is in Frame [2] that the following double inequality was also given:

5.3 $$ \frac{2+\cos x-\frac{x^{2}}{\pi^{2}}}{3-\frac{x^{2}}{\pi^{2}}}< \frac{ \sin x}{x}< \frac{2+\cos x-\frac{x^{2}}{10}}{3-\frac{x^{2}}{10}},\quad 0< x< \pi, $$

or

5.4 $$ \frac{2+\cos x}{3}-\frac{x(x-\sin x)}{3\pi^{2}}< \frac{\sin x}{x}< \frac{2+ \cos x}{3}- \frac{x(x-\sin x)}{30},\quad 0< x< \pi . $$

In order to compare the three inequalities (1.4), (5.2), and the right hand side of (5.4), we rewrite (1.4) as

5.5 $$ \frac{\sin x}{x}< \frac{1-\frac{x^{6}}{2100}}{1+\frac{(1-\cos x)^{2}}{9(3+2 \cos x)}}\frac{2+\cos x}{3}. $$

(i) We first compare two inequalities (5.5) and (5.2) on the same interval $(0,\pi /2)$. We compute

$$\begin{aligned}& \biggl( 1+\frac{(1-\cos x)^{2}}{9(3+2\cos x)} \biggr) \biggl( \frac{2+ \cos x}{3}- \frac{1}{180}x^{4}+\frac{1}{3780}x^{6} \biggr) - \biggl( 1-\frac{x ^{6}}{2100} \biggr) \frac{2+\cos x}{3} \\& \quad = \frac{1}{170\text{,}100}\frac{\cos x+2}{2\cos x+3}i(x), \end{aligned}$$

where

$$ i(x)=-12\text{,}600\cos x-105x^{4}\cos x+59x^{6}\cos x-1470x^{4}+151x^{6}+6300 \cos^{2}x+6300. $$

Numerical results show that $i(x)>0$ for all $x\in (0, 0.0040)$ and $i(x)<0$ for all $x\in (0.0040,\pi /2)$. That is, the upper estimate in (5.5) is smaller than the one in (5.2) on the interval $(0,0.0040)$, meanwhile the upper estimate in (5.2) is smaller than the one in (5.5) on the interval $(0.0040,\pi /2)$. So these two inequalities (1.4) and (5.2) are not included in each other.

(ii) Then we compare the two inequalities (5.5) and the right hand side of (5.4) on the same interval $(0,\pi )$. Let us check the function

$$\begin{aligned} \begin{aligned} &\frac{x^{6}}{2100}\frac{2+\cos x}{3}+\frac{(1-\cos x)^{2}}{9(3+2 \cos x)}\frac{2+\cos x}{3}- \frac{x(x-\sin x)}{30} \biggl( 1+\frac{(1- \cos x)^{2}}{9(3+2\cos x)} \biggr) \\ &\quad = \frac{1}{18\text{,}900}\frac{\cos x+2}{2\cos x+3}j(x), \end{aligned} \end{aligned}$$

where

$$\begin{aligned} j(x) &=-1400\cos x-70x^{2}\cos x+6x^{6}\cos x+980x\sin x-980x^{2}+9x ^{6} \\ &\quad {}+700\cos^{2}x+70x\cos x\sin x+700. \end{aligned}$$

Numerical results show that $j(x)>0$ for all $x\in (0, 0.4878)$ and $i(x)<0$ for all $x\in (0.4878,\pi )$. That is, the upper estimate in (5.5) is smaller than the one in the right hand side of (5.4) on the interval $(0,0.4878)$, meanwhile the upper estimate in the right hand side of (5.4) is smaller than the one in (5.5) on the interval $(0.4878,\pi )$. So these two inequalities (1.4) and the right hand side of (5.4) are not included in each other.

In a word, inequality (1.4) is not contained in the other improved Cusa–Huygens inequalities showed in [14] and [2] and is stronger than those ones near $x=0$.

### Remark 3

Using the methods in [15–17] and in [18], one can directly prove the inequalities (1.3) and (1.4), (1.5) and (1.6), respectively. A different approach based on the power series expansions, to proving, refinements and generalizations of inequalities of the similar type can be found in [19].

## Conclusions

In the present study, we find that there are two wrong inequalities for circular functions in the famous monograph “Analytic Inequalities” by Mitrinovic, and we reestablish two inequalities on this topic and create two corresponding inequalities for hyperbolic functions. These new inequalities are the generalization of the famous Cusa–Huygens inequality, one of them is not contained in other improved Cusa–Huygens inequalities showed in [14] and [2] and is stronger than the ones near $x=0$.
